# Postmortem submersion interval prediction model based on the rat muscle microbiome

**DOI:** 10.3389/fmicb.2025.1685097

**Published:** 2026-01-08

**Authors:** Cheng-Dong Ma, Xin-Biao Liao, Jia-Cheng Yue, Zhen Gao, Shu-Quan Zhao, Er-Wen Huang, Hu Zhao

**Affiliations:** 1 Faculty of Forensic Medicine, Zhongshan School of Medicine, Sun Yat-sen University, Guangzhou, China; 2 Guangdong Public Security Judicial Appraisal Center, Guangzhou, China; 3 Guangdong Province Translational Forensic Medicine Engineering Technology Research Center, Sun Yat-sen University, Guangzhou, China; 4 Guangzhou Nansha District Material Evidence Identification Center, Guangzhou, China

**Keywords:** forensic microbiology, machine learning, microbiota, post-mortem submersion interval, skeletal muscle

## Abstract

**Objective:**

Accurate estimation of post-mortem submersion interval (PMSI) is a critical challenge in forensic science. The current research has largely focused on the microbial communities in the skin and gut, which are susceptible to environmental contamination, while the potential of internal tissues remains underexplored. This study aimed to investigate whether the microbiome of skeletal muscle, a relatively closed ecosystem, undergoes a predictable succession following submersion in water PMSI and to evaluate its potential for building a high-precision PMSI prediction model, which is independent of the cause of death (drowning vs. post-mortem submersion).

**Methods:**

Using 72 male Sprague–Dawley rats, we established drowning (D group) and post-mortem submersion (PS group) models. After submersion in natural aquatic environment for 14 days, skeletal muscle samples were collected at six time. The microbial communities were profiled by high-throughput sequencing of the V3–V4 region of the 16S rRNA gene, followed by analyses of alpha and beta diversity. Based on the observed successional patterns, a two-stage prediction model combining classification and regression algorithms (e.g., random forest, RF) was developed.

**Results:**

The skeletal muscle microbiome exhibited a significant and predictable successional pattern, clearly partitioning into an early-phase (0–3 days) and a late-phase (5–14 days) (PERMANOVA, *p* < 0.001). This succession was characterized by a shift from the dominant community of Proteobacteria to the dominant community of Firmicutes. Importantly, the cause of death did not significantly impact either the alpha or beta diversity of the microbial communities (PERMANOVA, *p* = 0.251). The resulting two-stage prediction model demonstrated excellent performance: the classification model distinguished the early and late phases with an accuracy of 90.9% (AUC = 0.9504), and the mean absolute errors (MAE) of regression models was 0.303 days in the early phase and 1.293 days in the late phase.

**Conclusion:**

The rat skeletal muscle microbiome undergoes a regular and predictable post-mortem succession unrelated to the cause of death. The stable “microbial clock” within the internal tissue allows the construction of a high-precision two-stage machine learning model for PMSI estimation. Our results establish skeletal muscle as a highly promising new target for forensic microbiology, offering a robust theoretical basis and technical approach to resolving challenges in long-term PMSI estimation.

## 1 Introduction

The accurate estimation of the post-mortem submersion interval (PMSI) is a cornerstone of forensic science practice, providing critical information for criminal investigations, suspect validation, and event timeline reconstruction (van Daalen et al., 2017; García et al., 2021). Traditional methods, such as assessing physical changes like body temperature, rigor mortis, and livor mortis, are primarily applicable to the early post-mortem period (typically within 72 h) and their accuracy is easily compromised by various factors including ambient temperature and individual variations (Pittner et al., 2020; Zissler et al., 2020). Furthermore, forensic entomology provides limited information for PMSI estimation of submerged remains, as colonization by terrestrial insects typically only occurs once the cadaver floats to the surface. Consequently, the development of objective, universally applicable methods for estimating longer-term PMSI remains a significant challenge and an urgent need in the forensic field.

In recent years, the advent of high-throughput sequencing technologies has catalyzed the emergence of forensic microbiology, opening a novel frontier for PMSI estimation. A substantial body of research has demonstrated that upon the host’s death, its symbiotic microbial communities, both internal and external, undergo dynamic and predictable successional changes (Metcalf et al., 2013; Metcalf et al., 2016). This concept of a “microbial clock” has been validated in various animal models and human cadaver studies. Current research has predominantly focused on communities in the gut (Zhang et al., 2022; DeBruyn and Hauther, 2017), oral cavity (Metcalf et al., 2017), and on the skin (Lax et al., 2015), collectively establishing a strong time-dependent relationship between microbial succession and PMSI and enabling the development of preliminary prediction models using machine learning algorithms (Liu et al., 2020).

However, despite this significant progress, two core challenges hinder the translation of these findings into routine forensic practice. First, the focus on the gut and body surfaces—sites in direct contact with the external environment—means that these microbial communities are highly susceptible to random invasion by environmental microbes, contamination from the surrounding medium (e.g., water, soil), and disturbances from insect activity, all of which can compromise the stability and accuracy of the “microbial clock” (Bone et al., 2024; Wallace et al., 2021). Second, the specific impact of the cause of death on microbial succession remains largely unclear. Different manners of death, such as acute trauma versus drowning, may result in vastly different initial physiological states (e.g., circulatory status, tissue oxygenation), but whether this systematically alters the subsequent trajectory of microbial succession remains an unresolved question.

To address these knowledge gaps, this study focuses on a long-overlooked yet unique target for forensic microbiology: skeletal muscle. As an internal tissue with immense biomass that is relatively enclosed during the early stages of decomposition, skeletal muscle may provide a more stable source of information for PMSI estimation, one less affected by random external factors. We, therefore, hypothesized that the microbial community within skeletal muscle undergoes a regular and predictable succession following submersion in water (PMSI) and that this internal successional pattern remains highly consistent regardless of the cause of death (drowning vs. post-mortem submersion). In this study, we employed a controlled rat model to systematically track the dynamic changes in the skeletal muscle microbiome over a 14-day submersion period. By integrating machine learning algorithms, we aimed to answer the following questions: (1) Does a clear, time-dependent successional pattern exist in the skeletal muscle microbiome? (2) Does the manner of entry into the water significantly affect this pattern? (3) Can a PMSI prediction model based on skeletal muscle microbial data achieve the accuracy required for forensic applications? Our results reveal a distinct succession characterized by a shift from Proteobacteria to Firmicutes dominance and demonstrate the successful construction of a high-precision, two-stage PMSI prediction model that is robust to the cause of death, offering a novel and potent solution for forensic PMSI estimation.

## 2 Materials and methods

### 2.1 Animal model and experimental design

All animal experiments were conducted in strict accordance with animal welfare and ethical guidelines and were reviewed and approved by the Institutional Animal Care and Use Committee of Guangzhou Hua Teng Biotechnology Co., Ltd. (Approval No. B202503-1). This study utilized 72 healthy adult male Sprague–Dawley (SD) rats, aged 8 weeks, sourced from Zhuhai BesTest Bio-Tech Co., Ltd. All rats were acclimatized for 1 week in a barrier facility (temperature 20–26 °C, relative humidity 40–70%, 12 h/12 h light/dark cycle, pressure differential ≥10 Pa, air changes ≥15 per hour), with ad libitum access to irradiated standard chow (Jiangsu Co-Shine Bio-Tech Co., Ltd.) and autoclaved drinking water.

To investigate the influence of the cause of death on post-mortem microbial succession, the 72 rats were randomly assigned to either a drowning group (D, *n* = 36) or a post-mortem submersion group (PS, *n* = 36). The experiment was conducted under natural outdoor conditions with a recorded ambient temperature ranging from 24.8 °C to 29.6 °C. To ensure environmental consistency, the D group and PS group rats were housed in two separate large transparent water tanks, filled with natural water to a depth of over 30 cm. For the D group, rats were placed in mesh bags and subjected to repeated cycles of submersion and air exposure to simulate drowning until vital signs ceased. For the PS group, rats were euthanized via CO₂ inhalation. Following confirmation of death, carcasses from both groups, still within their mesh bags, were fully submerged in the water tanks, with each carcass housed individually. To observe the dynamic changes, both the D and PS groups were further divided into six temporal subgroups corresponding to post-mortem submersion intervals of 0, 1, 3, 5, 7, and 14 days, with each time point containing six biological replicates.

### 2.2 Sample collection and preservation

At each designated time point, all six rats from the corresponding subgroup were retrieved for sample collection. All procedures were performed under sterile conditions, using a separate set of autoclaved dissection instruments for each animal. The skin and fascia of the hind limb were aseptically incised to expose the quadriceps or gastrocnemius muscle. A tissue block of approximately 0.5–1.0 g was excised from the deep central region of the muscle. All collected samples were immediately placed in pre-labeled, cryo-resistant screw-cap tubes, flash-frozen in liquid nitrogen, and subsequently stored at −80 °C until DNA extraction.

### 2.3 Microbiome DNA extraction and 16S rDNA sequencing

Total microbial DNA was extracted from approximately 0.5–1.0 mg of each skeletal muscle samples using the Bacterial Genomic DNA Extraction Kit (DP302-02, Tiangen Biotech (Beijing) Co., Ltd.), strictly following the manufacturer’s protocol. The concentration and purity of the extracted DNA were quantified using a Qubit^®^ 4.0 Fluorometer (Invitrogen, United States) with the dsDNA HS Assay Kit and assessed for integrity via 1% agarose gel electrophoresis.

The V3–V4 hypervariable region of the bacterial 16S rRNA gene was amplified using the primers 341F (5′-CCTACGGGNGGCWGCAG-3′) and 805R (5′-GACTACHVGGGTATCTAATCC-3′), synthesized by Sangon Biotech (Shanghai) Co., Ltd. PCR was performed in a 25 μL reaction volume containing 12.5 μL of Phusion Hot start flex 2X Master Mix, 2.5 μL of each 10 μM primer, and 50 ng of template DNA. The thermal cycling conditions were: an initial denaturation at 98 °C for 30 s; followed by 32 cycles of 98 °C for 10 s, 54 °C for 30 s, and 72 °C for 45 s; and a final extension at 72 °C for 10 min.

Amplified PCR products were verified by 2% agarose gel electrophoresis and purified using AMPure XT beads (Beckman Coulter Genomics, United States). The quality of the resulting libraries was assessed with an Agilent 2100 Bioanalyzer (Agilent, United States). Qualified libraries were pooled in equimolar amounts and sequenced on an Illumina NovaSeq 6000 platform using a NovaSeq 6000 SP Reagent Kit (500 cycles) for PE250 paired-end sequencing. This sequencing service was provided by LC-Bio Technology Co., Ltd. (Hangzhou, China).

### 2.4 Bioinformatic and statistical analysis

Raw sequencing data were processed using the QIIME2 pipeline (v2019.7). After demultiplexing, primers and barcodes were removed using the cutadapt plugin. The DADA2 plugin was then employed for sequence denoising, merging of paired-end reads, chimera removal, and generation of an amplicon sequence variant (ASV) feature table. The amplified V3–V4 region resulted in merged sequences of approximately 460 bp. A total of 7,587,720 high-quality reads were obtained from all samples after filtering. Rarefaction curves confirmed that the sequencing depth was sufficient to capture the majority of microbial diversity in each sample (Supplementary Figure 1). Taxonomic classification of ASVs was performed against the SILVA database (Release 138).

All statistical analyses were conducted in R (v4.1.3). Alpha diversity indices (Chao1, Shannon) and beta diversity metrics (Bray–Curtis) were calculated using the vegan and phyloseq packages. The Wilcoxon rank-sum test was used to compare alpha diversity between groups. Permutational multivariate analysis of variance (PERMANOVA) was employed to test for significant differences in community structure. Machine learning models were constructed using the randomForest package, with the relative abundances of microbial genera as predictor variables and the PMSI as the response variable. The performance of classification models was evaluated by the area under the receiver operating characteristic (ROC) curve (AUC), while regression model performance was assessed by the mean absolute error (MAE). A *p*-value <0.05 was considered statistically significant for all tests.

## 3 Results

### 3.1 Dynamic changes in the taxonomic composition of the microbial community

**Figure 1:**
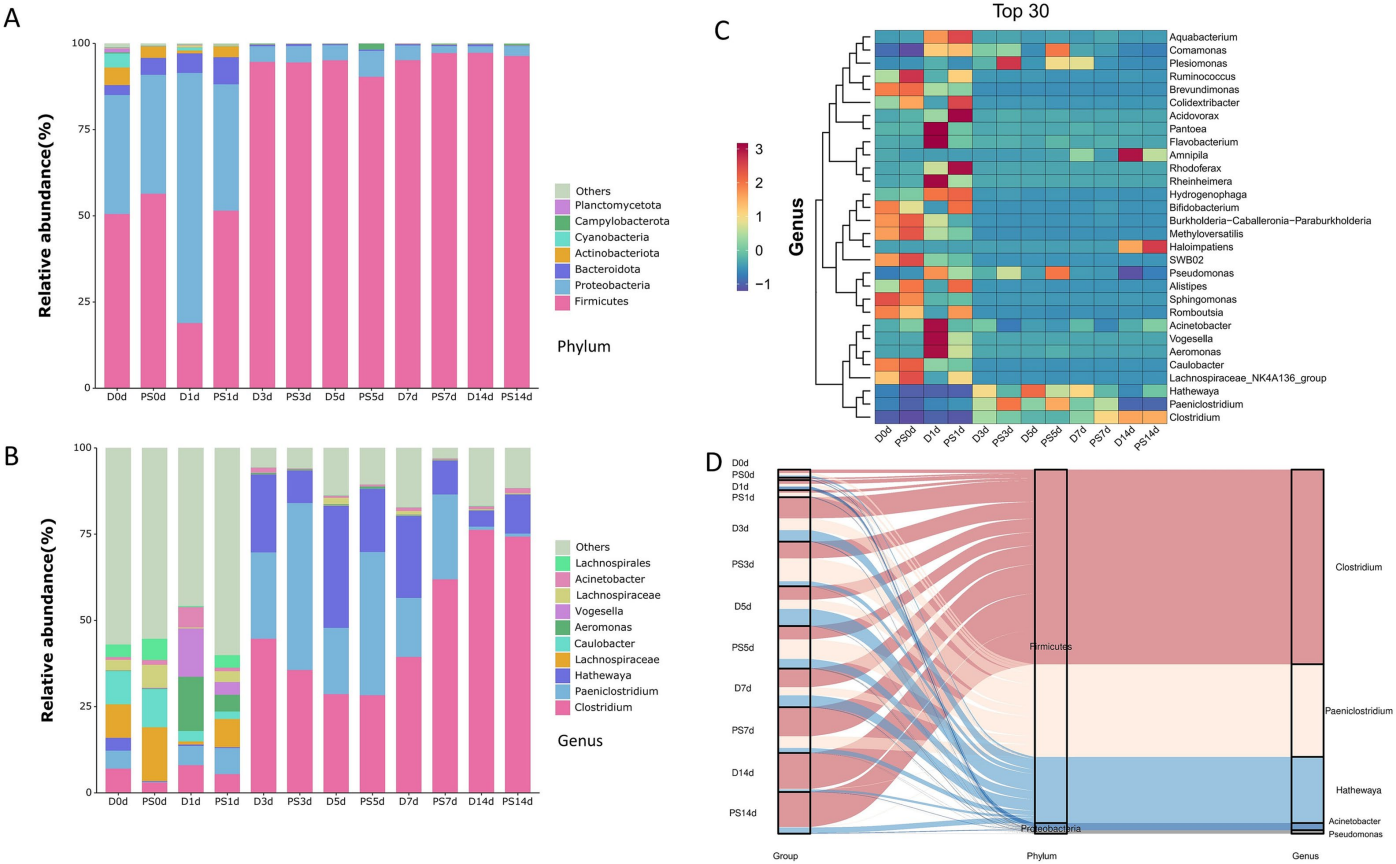
Compositional dynamics and successional patterns of the muscle microbial community at different taxonomic levels. **(A)** Stacked bar chart showing the relative abundance of dominant bacterial phyla over the 14-day PMSI. **(B)** Stacked bar chart displaying the relative abundance of dominant bacterial genera. The legend shows the top 10 most abundant genera. **(C)** Heatmap illustrating the *Z*-score normalized relative abundance of the top 30 most abundant genera across all samples. Samples are ordered by PMI in days, and genera are clustered hierarchically based on their abundance profiles. **(D)** Sankey diagram illustrating the compositional flow from sample groups (ordered by PMSI) to phylum and genus levels. The width of the flows is proportional to the relative abundance.

To identify the key microbial taxa driving community succession, we analyzed the relative abundance of species at different taxonomic levels. At the phylum level, the microbial community composition underwent a systematic change with increasing post-mortem submersion interval (PMSI). At the initial time point (day 0), the community was primarily composed of Firmicutes and Proteobacteria. As time progressed, the relative abundance of Proteobacteria consistently increased, rising from less than 20% initially to over 60% by day 14, while the relative abundance of Firmicutes steadily decreased (Figure 1A).

At the genus level, we observed more detailed successional patterns. A heatmap analysis revealed two main successional clusters based on the hierarchical clustering of genus abundance patterns (Figure 1C). During the early decomposition phase (0–3 days), genera such as *Pseudomonas*, *Acinetobacter*, and *Vogesella* exhibited higher relative abundance. As decomposition progressed into the late phase (5–14 days), the abundance of these early dominant genera significantly declined, while that of *Clostridium* and *Paeniclostridium* sharply increased, making them the dominant members of the late-stage community. This pattern was also confirmed by the stacked bar charts of genus-level composition (Figure 1B). A Sankey diagram further visualized this cross-level association, showing that in late-stage samples, the increased abundance of the phylum Firmicutes (pink flow) was almost entirely attributed to *Clostridium* and *Paeniclostridium*, whereas the early-stage Proteobacteria (blue flow) was composed of several different genera (Figure 1D). To assess the potential influence of the surrounding water on the muscle microbiome, we compared the microbial genera shared between water and muscle samples collected at day 0 and day 14. The analysis revealed distinct dynamics between the two groups (Supplementary Figure 2). In the post-mortem submersion (PS) group, the number of shared genera increased from 64 at day 0 to 111 at day 14. In contrast, the drowning (D) group exhibited a high initial number of shared genera (112) at day 0, which remained at a similarly high level (105) by day 14.

### 3.2 Limited impact of cause of death on microbial community diversity

**Figure 2:**
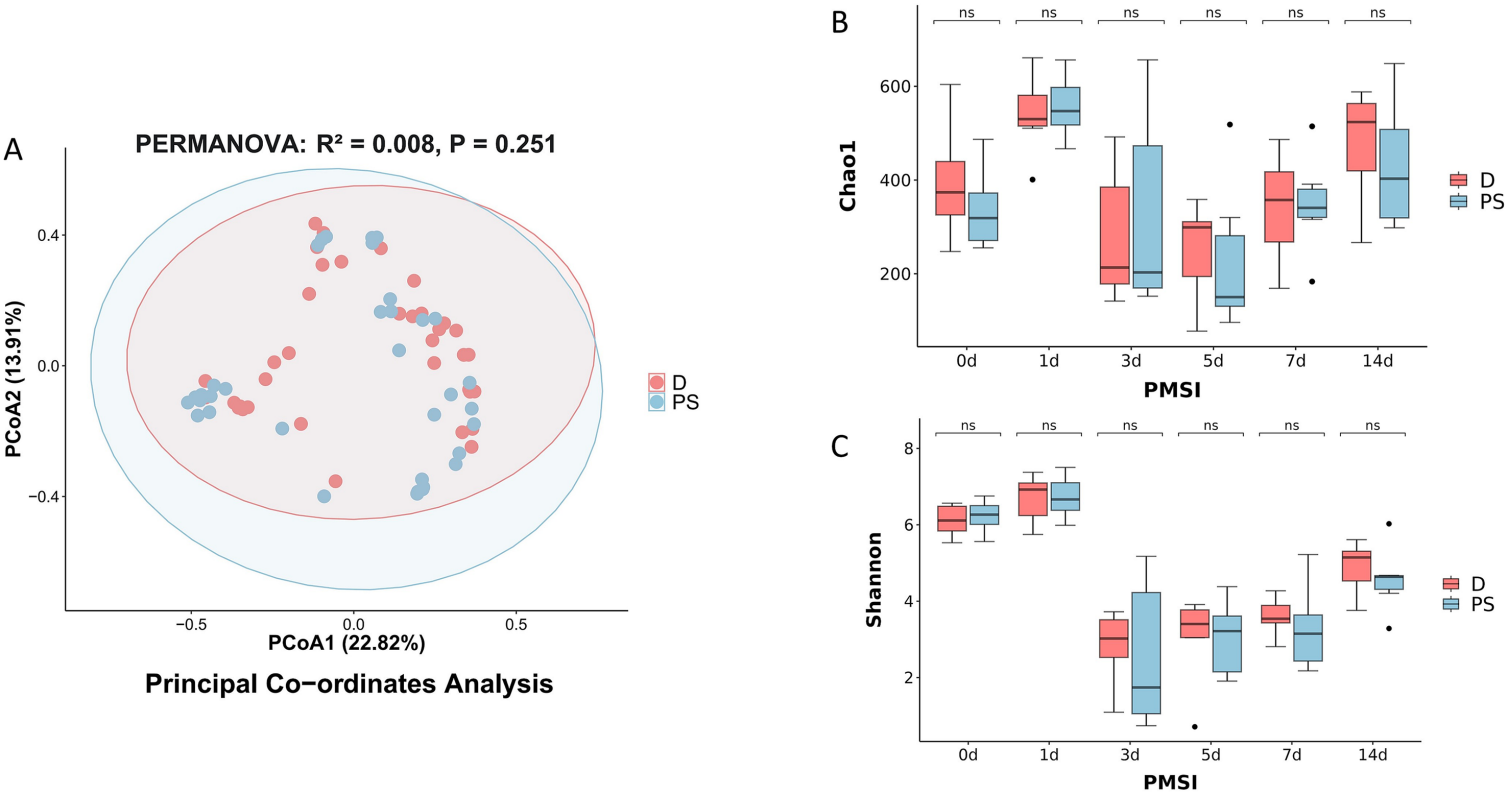
Comparison of muscle microbial community diversity between the drowning (D) and post-mortem submersion (PS) groups. **(A)** Principal co-ordinates analysis (PCoA) plot based on Bray–Curtis dissimilarity, illustrating the overall microbial community structure. The first two principal coordinates explain 22.82 and 13.91% of the total variation, respectively. Each point represents an individual sample, colored by group (D: red; PS: blue). Ellipses indicate the 95% confidence interval for each group. **(B,C)** Boxplots comparing the alpha diversity between the D and PS groups at each PMSI. Community richness was estimated using the Chao1 index **(B)**, and community diversity was estimated using the Shannon index **(C)**. No significant differences (ns, *p* > 0.05) were observed between the two groups at any time point according to the Wilcoxon rank-sum test. The boxes represent the interquartile range (IQR), the central line indicates the median, and the whiskers extend to 1.5 times the IQR.

To investigate whether the manner of entry into the water was a key factor influencing the microbial succession in skeletal muscle, we directly compared the community structure and diversity of the drowning (D) and post-mortem submersion (PS) groups. A principal co-ordinates analysis (PCoA) based on the Bray–Curtis distance showed that the sample points from the D and PS groups were highly intermixed in the ordination space, without forming separate, distinct clusters. The 95% confidence ellipses for the two groups also largely overlapped (Figure 2A). To statistically validate this observation, we performed a permutational multivariate analysis of variance (PERMANOVA). The results confirmed that there was no significant difference in the overall community structure (beta diversity) between the two groups (PERMANOVA, *R*^2^ = 0.008, *p* = 0.251). The *R*^2^ value indicated that the cause of death could only explain 0.8% of the total community variation.

We further assessed the alpha diversity of the communities. The results showed that for both the Chao1 index, which measures species richness (Figure 2B), and the Shannon index, which measures both richness and evenness (Figure 2C), the boxplot distributions for the D and PS groups were highly similar at all examined time points (0, 1, 3, 5, 7, and 14 days). A Wilcoxon rank-sum test confirmed that at no time point was there a statistically significant difference in any alpha diversity index between the two groups (all *p* > 0.05). Taken together, the results from both beta and alpha diversity analyses indicate that, under the conditions of this study, the cause of death (drowning vs. post-mortem submersion) was not a major driver shaping the successional patterns of the skeletal muscle microbiome.

### 3.3 Stage-wise division of microbial community succession based on time

**Figure 3:**
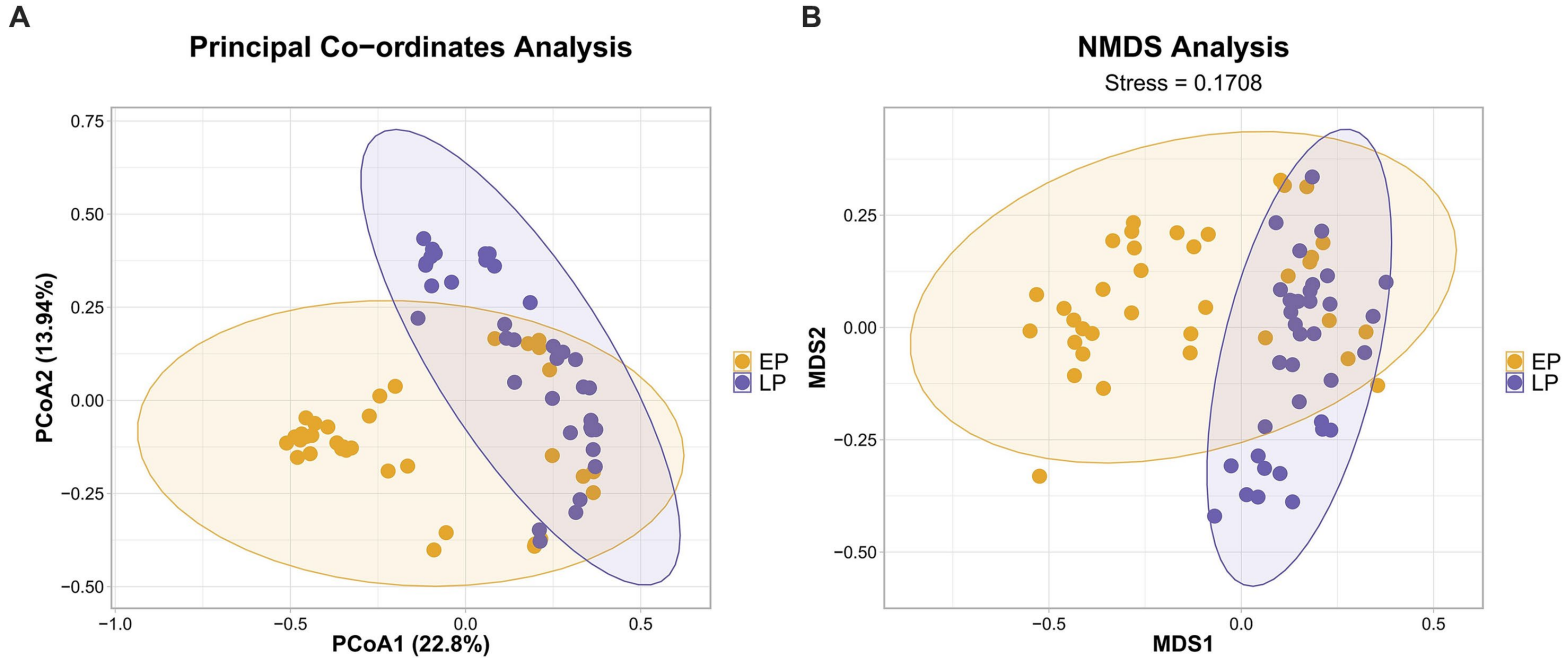
Distinct microbial community structures in rat muscle at early and late post-mortem submersion intervals. **(A)** Principal co-ordinates analysis (PCoA) and **(B)** non-metric multidimensional scaling (NMDS) plots illustrating the beta-diversity of microbial communities, based on the Bray–Curtis dissimilarity metric. The first two principal coordinates in the PCoA plot explain 22.8 and 13.94% of the total variation, respectively. The NMDS plot has a stress value of 0.1708. Each point represents an individual sample. Samples are colored according to their post-mortem submersion interval (PMSI) phase: yellow for the early-phase (EP; 0–3 days) and purple for the late-phase (LP; 5–14 days). Ellipses represent the 95% confidence interval for each group. The separation between the two phases was statistically significant (PERMANOVA, *p* < 0.001).

Having established that the cause of death had a limited impact, we analyzed all samples together to investigate whether the microbial community exhibited stage-wise successional characteristics. A PCoA based on the Bray–Curtis distance matrix revealed that all sample points were distributed along a clear temporal gradient, primarily driven by the first principal coordinate (PCoA1), which explained 22.8% of the community variation (Figure 3A). To further quantify this successional pattern, we divided the PMSI into an early-phase (EP; 0–3 days) and a late-phase (LP; 5–14 days). Both PCoA and non-metric multidimensional scaling (NMDS) analysis (stress = 0.1708) consistently showed that the samples from these two phases formed two statistically distinct clusters (PERMANOVA, *p* < 0.001), with their 95% confidence ellipses showing minimal overlap in the ordination space (Figure 3B).

### 3.4 PMSI prediction based on a stage-wise succession model

**Figure 4:**
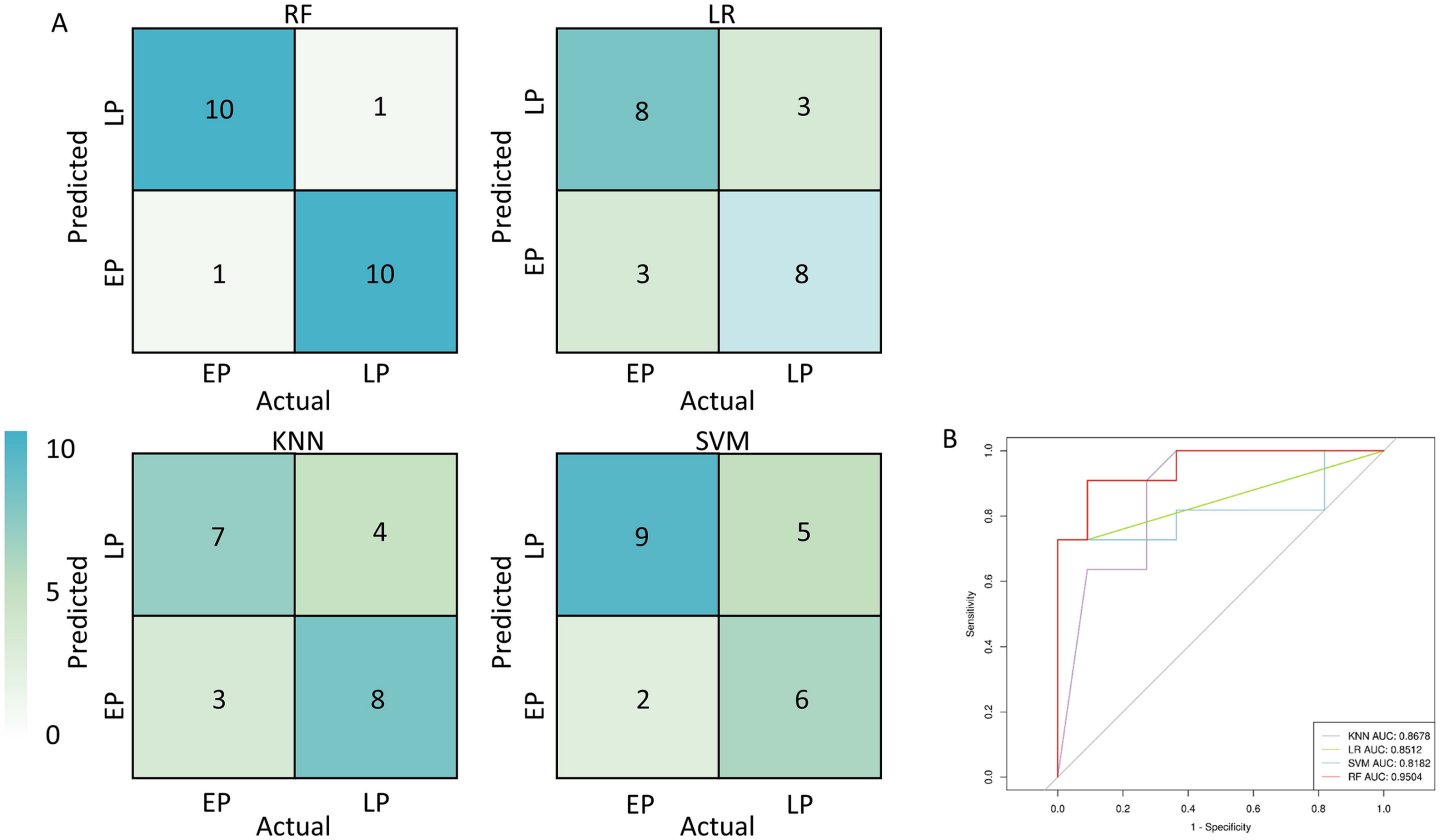
Performance evaluation of machine learning models for classifying early-phase (EP) and late-phase (LP) post-mortem submersion intervals. **(A)** Confusion matrices for four different classification models: random forest (RF), logistic regression (LR), *k*-nearest neighbors (KNN), and support vector machine (SVM). The values in each cell represent the number of samples in the test set. The color intensity corresponds to the number of samples. **(B)** Receiver operating characteristic (ROC) curves for the four models. The area under the curve (AUC) for each model is displayed in the legend.

Based on the clear stage-wise succession of the microbial community, we evaluated the performance of several machine learning algorithms in classifying samples into the early-phase (EP, 0–3 days) and late-phase (LP, 5–14 days). We constructed four classification models: random forest (RF), logistic regression (LR), *k*-nearest neighbors (KNN), and support vector machine (SVM). By comparing their confusion matrices and receiver operating characteristic (ROC) curves, we found that the RF model exhibited the best classification performance (Figure 4A). Specifically, on an independent test set of 22 samples, the RF model correctly predicted 20 samples, achieving an overall accuracy of 90.9%. Its area under the ROC curve (AUC) was the highest among the four models, reaching 0.9504 (Figure 4B).

**Figure 5:**
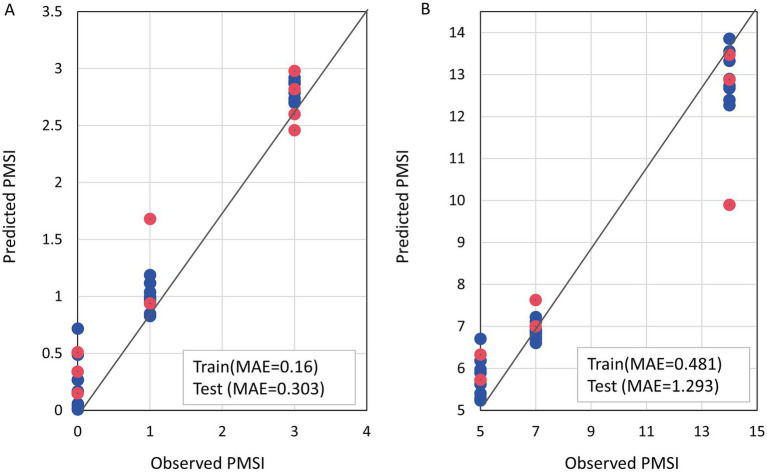
Performance of the two-stage random forest regression models for PMSI prediction. Scatter plots showing the correlation between the observed PMSI (in days) and the PMSI predicted by the regression models for **(A)** the early-phase (EP; 0–3 days) and **(B)** the late-phase (LP; 5–14 days). Each point represents an individual sample, colored by its inclusion in either the training set (blue) or the test set (red). The solid black line represents the ideal prediction line (*y* = *x*). The mean absolute error (MAE) for both the training and test sets is displayed in the inset box for each model.

After successfully establishing the stage classification, we built separate RF regression models for the EP and LP phases to accurately predict the PMSI. For the EP group (0–3 days), the model demonstrated extremely high prediction accuracy. As shown in Figure 5A, the sample points for both the training and test sets were tightly clustered around the ideal prediction line (*y* = *x*). The model’s mean absolute error (MAE) on the training set was 0.16 days, and on the independent test set, the MAE was only 0.303 days. For the more complex LP group (5–14 days), the regression model’s predictions were also robust. The scatter plot (Figure 5B) showed a strong correlation between the predicted and actual values for most samples. The MAE for this model was 0.481 days on the training set and 1.293 days on the test set.

## 4 Discussion

This study aimed to investigate whether the microbial community of skeletal muscle, a relatively closed internal ecosystem, undergoes a predictable succession following submersion in water (PMSI) and to evaluate its utility for forensic PMSI estimation. Our central finding confirms that the rat skeletal muscle microbiome undergoes a highly predictable, stage-wise succession, enabling the construction of a two-stage machine learning model that accurately predicts PMSI up to 14 days. These results open a new avenue in forensic microbiology by highlighting the potential of internal tissues as stable sources of information for PMSI estimation.

Our research first established the overall pattern of microbial succession in skeletal muscle. Beta diversity analysis clearly showed a significant temporal gradient in community structure, which could be distinctly partitioned into an early (0–3 days) and a late (5–14 days) phase (Figure 3). This finding is consistent with previous studies on traditional sample sites like the gut and skin, which also report time-dependent microbial succession as the biological basis for the “microbial clock” (Metcalf et al., 2013; Guo et al., 2016). The novelty of our work, however, lies in systematically demonstrating this principle within the relatively “sterile” environment of skeletal muscle. This approach complements other sampling strategies aimed at estimating PMSI from skeletal remains. For instance, Rose et al. (2025) demonstrated the efficacy of using surface swabs, a valuable non-destructive method. Our focus on a well-protected internal tissue like skeletal muscle is intended to capture a more stable, endogenously-driven successional signal, thereby potentially reducing environmental stochasticity. We speculate that this phased transition marks a critical ecological turning point in the decomposition process. The early phase may be dominated by facultative anaerobes that spread via the circulatory system after death, while the late phase, characterized by increased tissue hypoxia and structural breakdown, provides an ecological niche for the proliferation of obligate anaerobes, particularly various *Clostridium* species.

At the taxonomic level, we observed a significant shift from Proteobacteria to Firmicutes dominance, specifically a succession from genera like *Pseudomonas* to *Clostridium* (Figure 1). This pattern not only corroborates the ecological shift mentioned above at a species level but also shares commonalities with findings from classic terrestrial decomposition models. For instance, during the bloat and active decay stages of terrestrial decomposition, obligate anaerobic *Clostridium* species are widely recognized as the core microbes driving tissue breakdown and gas production (Metcalf et al., 2016; Cobaugh et al., 2015). Our data suggest that even in the cold and humid conditions of an aquatic environment, the internal microenvironment of skeletal muscle (e.g., hypoxia) may still be the primary intrinsic driver of core microbial succession, with its underlying logic bearing some resemblance to that in terrestrial environments. Notably, the Sankey diagram (Figure 1D) visually demonstrates that the increase in Firmicutes in the late stage is almost entirely contributed by *Clostridium* and *Paeniclostridium*, a highly focused successional pattern that may offer promising candidates for biomarkers of long-term PMSI.

A key finding of this study is that the manner of entry into the water (drowning vs. post-mortem submersion) did not have a statistically significant effect on either the alpha or beta diversity of the skeletal muscle microbiome (Figure 2). This result may seem counterintuitive, as the drowning process theoretically introduces a large number of aquatic microbes into the respiratory and circulatory systems. A possible explanation is that skeletal muscle, as a well-vascularized but relatively closed tissue, presents a strong “ecological barrier” due to its robust anaerobic microenvironment and specific nutrient conditions. This barrier may make it difficult for typically aerobic or facultative anaerobic microbes from the water to effectively colonize and proliferate, thus failing to disrupt the overall successional trajectory dominated by endogenous bacteria. This finding has significant practical implications, suggesting that PMSI estimation models based on skeletal muscle may have greater stability than those based on surface microbes, being less affected by case-specific details such as the manner of death. This is consistent with the findings of Zhang et al. (2022) in the mouse gut, where they also observed that the cause of death had a limited impact on the succession of the gut microbiome.

The microbial exchange dynamics between water and muscle offered key insights. In the PS group, the initial sharing of 64 genera likely represents low-abundance transient and background taxa rather than significant colonization. More intriguingly, the decrease in shared taxa in the D group (from 112 to 105) suggests a process of ecological selection. We hypothesize that the selective internal microenvironment favors endogenous anaerobes that outcompete the large microbial inoculum introduced during drowning. This compelling hypothesis of competitive exclusion warrants future investigation. Crucially, the resilience of the overall successional trajectory despite this major initial perturbation strongly supports the internal muscle microbiome as a robust target for PMSI estimation.

Based on these findings, we successfully constructed and validated a high-precision, two-stage PMSI prediction model. Our findings that microbial succession on submerged bone can be used to predict PMSI are highly consistent with the foundational work in this area. Notably, Cartozzo et al. (2021a) and Cartozzo et al. (2021b) have successfully developed similar random forest models based on the microbiome of *Sus scrofa* bones submerged in both freshwater lake and river environments, establishing the principle that bone is a reliable substrate for long-term PMSI estimation. By first using a classification model to accurately determine the successional stage of a sample (AUC = 0.9504), and then applying a regression model optimized for that specific stage, we significantly improved the prediction accuracy (Figures 4, 5). Particularly in the early PMSI phase (0–3 days), the model’s mean absolute error was as low as 0.303 days, which is of great value for forensic applications. Even in the more complex late phase (5–14 days), the mean error of 1.293 days demonstrates its considerable potential as an auxiliary diagnostic tool. This “classify-then-regress” modeling strategy may be an effective paradigm for addressing the prediction challenges posed by the non-linear and stage-wise changes in microbial data, and is worth promoting in future forensic microbiology research (Yuan et al., 2023).

Despite the positive results of this study, several limitations exist. First, this study used an SD rat model, and there are physiological, immunological, and initial microbial compositional differences between rats and humans. Therefore, the direct applicability of the model to human cases still needs to be ultimately validated through research on other animal models (Yang et al., 2023). Second, the experiment was conducted under ambient temperature, and the lack of continuous water temperature monitoring prevented the calculation of accumulated degree-days (ADD). While the use of post-mortem submersion days served as a viable proxy in this exploratory study, we acknowledge that ADD is a more precise metric in forensic science. Future studies should prioritize the logging of water temperature to build more robust, ADD-based predictive models. Finally, this study focused primarily on the community structure of the microbiome and did not delve into the changes in its functional metabolism, the latter of which could provide deeper insights into the intrinsic mechanisms of succession and the discovery of more stable functional biomarkers.

## 5 Conclusion

In summary, this study is the first to systematically reveal the regular successional pattern of the rat skeletal muscle microbiome during post-mortem submersion in water, and successfully construct a high-precision two-stage PMSI prediction model. Our results not only show a novel and stable research goal for forensic microbiology but also provide a solid experimental basis and an innovative modeling approach for development of more reliable PMSI estimation tools. Future research should focus on verifying these findings in models closer to humans (e.g., pig models) and under more complex environmental conditions, and should be combined with macro-functional genomics and other methods. The ultimate goal is to transform this “microbial clock” into a powerful timing tool for forensic practice.

## Data Availability

The raw sequencing data for this study are available in the NCBI BioProject database under accession number PRJNA1370835.

## References

[ref1] Bone M. S. Legrand T. P. R. A. Harvey M. L. Wos-Oxley M. L. Oxley A. P. A. (2024). Aquatic conditions & bacterial communities as drivers of the decomposition of submerged remains. Forensic Sci. Int. 361:112072. doi: 10.1016/j.forsciint.2024.112072, 38838610

[ref2] Cartozzo C. Simmons T. Swall J. Singh B. (2021a). Postmortem submersion interval (PMSI) estimation from the microbiome of *Sus scrofa* bone in a freshwater river. Forensic Sci. Int. 318:110480. doi: 10.1016/j.forsciint.2020.110480, 33214010

[ref3] Cartozzo C. Singh B. Swall J. Simmons T. (2021b). Postmortem submersion interval (PMSI) estimation from the microbiome of *Sus scrofa* bone in a freshwater lake. J. Forensic Sci. 66, 1334–1347. doi: 10.1111/1556-4029.14692, 33818789

[ref4] Cobaugh K. L. Schaeffer S. M. DeBruyn J. M. (2015). Functional and structural succession of soil microbial communities below decomposing human cadavers. PLoS One 10:e0130201. doi: 10.1371/journal.pone.0130201, 26067226 PMC4466320

[ref5] DeBruyn J. M. Hauther K. A. (2017). Postmortem succession of gut microbial communities in deceased human subjects. PeerJ 5:e3437. doi: 10.7717/peerj.3437, 28626612 PMC5470579

[ref6] García M. D. Arnaldos M. I. Lago V. Ramírez M. Ubero N. Prieto J. . (2021). The paradigm of interdisciplinarity in forensic investigation. A case in Southeastern Spain. Leg. Med. 48:101817. doi: 10.1016/j.legalmed.2020.101817, 33264697

[ref7] Guo J. Fu X. Liao H. Hu Z. Long L. Yan W. . (2016). Potential use of bacterial community succession for estimating post-mortem interval as revealed by high-throughput sequencing. Sci. Rep. 6:24197. doi: 10.1038/srep24197, 27052375 PMC4823735

[ref8] Lax S. Hampton-Marcell J. T. Gibbons S. M. Colares G. B. Smith D. Eisen J. A. . (2015). Forensic analysis of the microbiome of phones and shoes. Microbiome 3:21. doi: 10.1186/s40168-015-0082-9, 25969737 PMC4427962

[ref9] Liu R. Gu Y. Shen M. Li H. Zhang K. Wang Q. . (2020). Predicting postmortem interval based on microbial community sequences and machine learning algorithms. Environ. Microbiol. 22, 2273–2291. doi: 10.1111/1462-2920.15000, 32227435

[ref10] Metcalf J. L. Wegener Parfrey L. Gonzalez A. Lauber C. L. Knights D. Ackermann G. . (2013). A microbial clock provides an accurate estimate of the postmortem interval in a mouse model system. eLife 2:e01104. doi: 10.7554/eLife.01104, 24137541 PMC3796315

[ref11] Metcalf J. L. Xu Z. Z. Bouslimani A. Dorrestein P. Carter D. O. Knight R. (2017). Microbiome tools for forensic science. Trends Biotechnol. 35, 814–823. doi: 10.1016/j.tibtech.2017.03.006, 28366290

[ref12] Metcalf J. L. Xu Z. Z. Weiss S. Lax S. Van Treuren W. Hyde E. R. . (2016). Microbial community assembly and metabolic function during mammalian corpse decomposition. Science 351, 158–162. doi: 10.1126/science.aad2646, 26657285

[ref13] Pittner S. Bugelli V. Weitgasser K. Zissler A. Sanit S. Lutz L. . (2020). A field study to evaluate PMI estimation methods for advanced decomposition stages. Int. J. Legal Med. 134, 1361–1373. doi: 10.1007/s00414-020-02278-0, 32248308 PMC7295721

[ref14] Rose S. Johnson H. Cartozzo C. Swall J. Simmons T. Singh B. (2025). Testing the efficacy of surface swab sampling to determine postmortem submersion interval (PMSI), using the microbiome colonization of skeletal remains. J. Forensic Sci. 70, 1261–1273. doi: 10.1111/1556-4029.70039, 40329496 PMC12223347

[ref15] van Daalen M. A. de Kat D. S. Oude Grotebevelsborg B. F. L. de Leeuwe R. Warnaar J. Oostra R. J. . (2017). An aquatic decomposition scoring method to potentially predict the postmortem submersion interval of bodies recovered from the North Sea. J. Forensic Sci. 62, 369–373. doi: 10.1111/1556-4029.13258, 28247448

[ref16] Wallace J. R. Receveur J. P. Hutchinson P. H. Kaszubinski S. F. Wallace H. E. Benbow M. E. (2021). Microbial community succession on submerged vertebrate carcasses in a tidal river habitat: implications for aquatic forensic investigations. J. Forensic Sci. 66, 2307–2318. doi: 10.1111/1556-4029.14869, 34462924

[ref17] Yang F. Zhang X. Hu S. Nie H. Gui P. Zhong Z. . (2023). Changes in microbial communities using pigs as a model for postmortem interval estimation. Microorganisms 11:2811. doi: 10.3390/microorganisms11112811, 38004822 PMC10672931

[ref18] Yuan H. Wang Z. Wang Z. Zhang F. Guan D. Zhao R. (2023). Trends in forensic microbiology: from classical methods to deep learning. Front. Microbiol. 14:1163741. doi: 10.3389/fmicb.2023.1163741, 37065115 PMC10098119

[ref19] Zhang F. Wang P. Zeng K. Yuan H. Wang Z. Li X. . (2022). Postmortem submersion interval estimation of cadavers recovered from freshwater based on gut microbial community succession. Front. Microbiol. 13:988297. doi: 10.3389/fmicb.2022.988297 36532467 PMC9756852

[ref20] Zissler A. Stoiber W. Steinbacher P. Geissenberger J. Monticelli F. C. Pittner S. (2020). Postmortem protein degradation as a tool to estimate the PMI: a systematic review. Diagnostics 10:1014. doi: 10.3390/diagnostics10121014, 33256203 PMC7760775

