# Supplementary material for: Postmortem submersion interval prediction model based on the rat muscle microbiome
Source: Front Microbiol. 2026 Jan 8;16:1685097. doi: 10.3389/fmicb.2025.1685097 (PMC12848373; doi:10.3389/fmicb.2025.1685097)
Supplement: Supplementary file 2 [file Data_Sheet_1.docx]

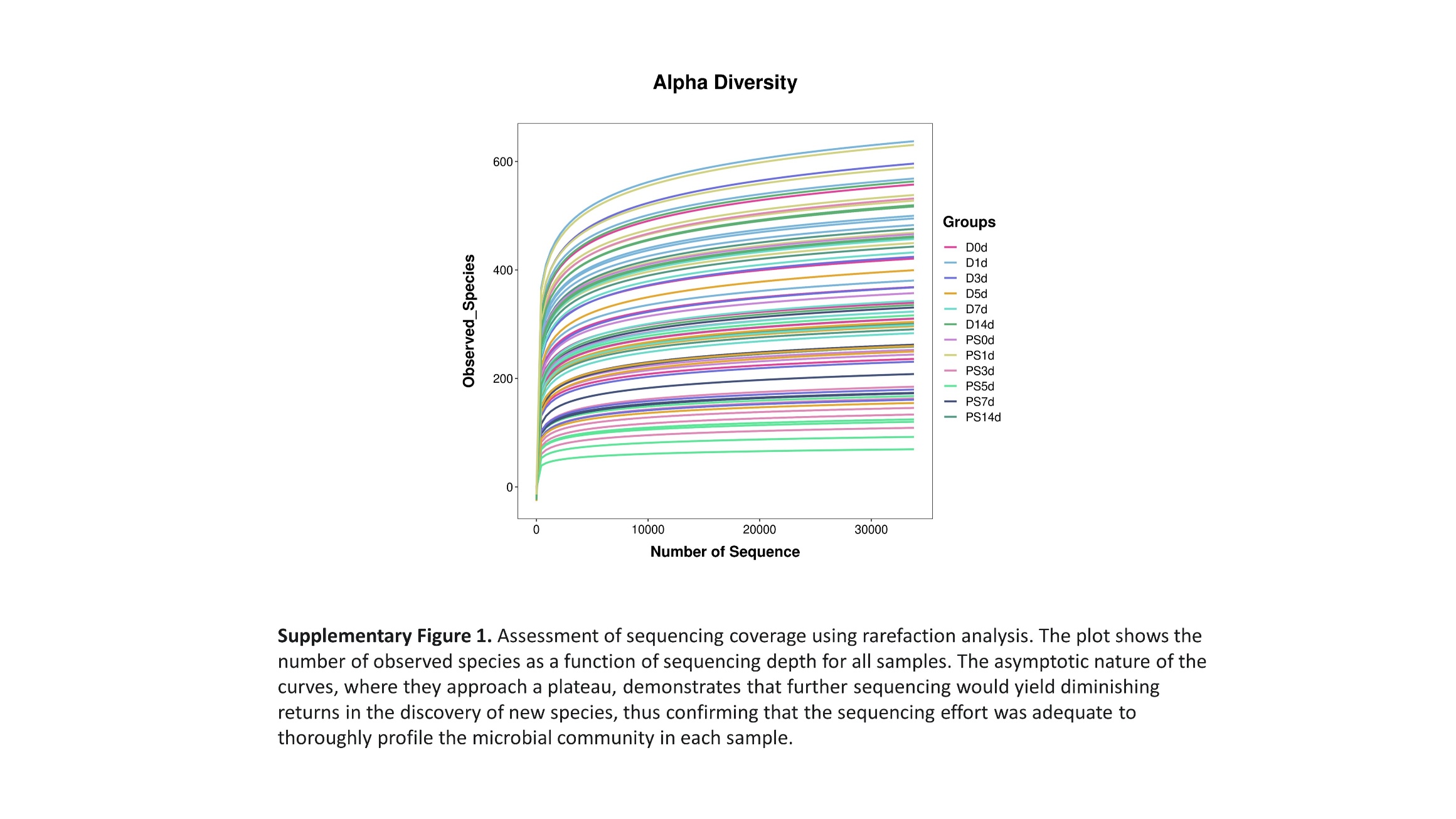


**Supplementary Figure 1.** Assessment of sequencing coverage using rarefaction analysis.

The plot shows the number of observed species as a function of sequencing depth for all samples. The asymptotic nature of the curves, where they approach a plateau, demonstrates that further sequencing would yield diminishing returns in the discovery of new species, thus confirming that the sequencing effort was adequate to thoroughly profile the microbial community in each sample.


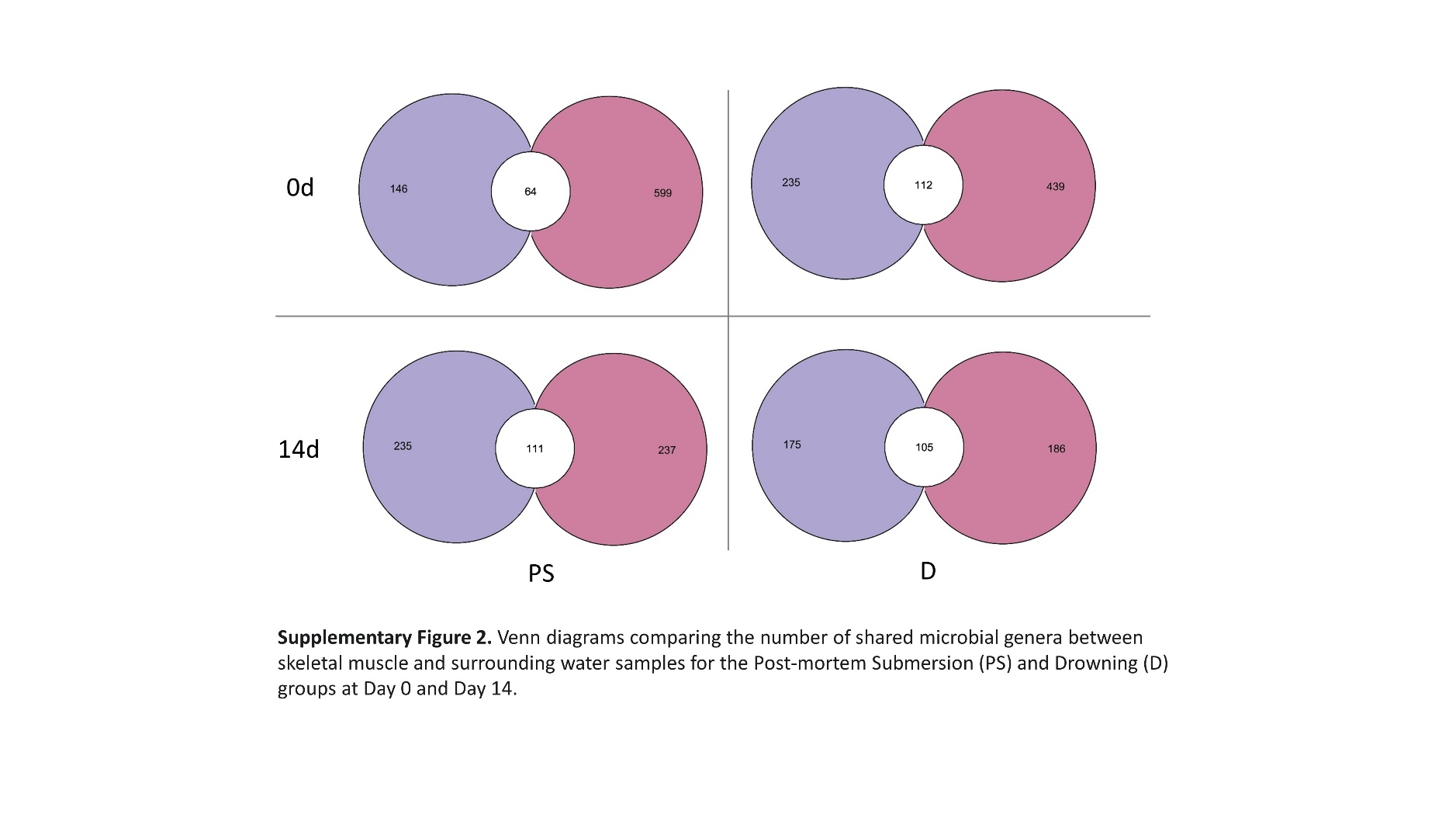


**Supplementary Figure 2.** Venn diagrams comparing the number of shared microbial genera between skeletal muscle and surrounding water samples for the Post-mortem Submersion (PS) and Drowning (D) groups at Day 0 and Day 14.
